# Extreme Polyploidy of *Carsonella*, an Organelle-Like Bacterium with a Drastically Reduced Genome

**DOI:** 10.1128/spectrum.00350-22

**Published:** 2022-04-18

**Authors:** Atsushi Nakabachi, Nancy A. Moran

**Affiliations:** a Electronics-Inspired Interdisciplinary Research Institute (EIIRIS), Toyohashi University of Technology, Toyohashi, Aichi, Japan; b Section of Integrative Biology, University of Texas, Austin, Texas, USA; University of Vienna

**Keywords:** ploidy, bacteriome-associated symbionts, small genome, insects

## Abstract

Polyploidy is the state of having multiple copies of the genome within a nucleus or a cell, which has repeatedly evolved across the domains of life. Whereas most bacteria are monoploid, some bacterial species and endosymbiotic organelles that are derived from bacteria are stably polyploid. In the present study, using absolute quantitative PCR, we assessed the ploidy of Candidatus Carsonella ruddii (Gammaproteobacteria, Oceanospirillales), the obligate symbiont of the hackberry petiole gall psyllid, Pachypsylla venusta (Hemiptera, Psylloidea). The genome of this symbiont is one of the smallest known for cellular organisms, at 160 kb. The analysis revealed that *Carsonella* within a single bacteriocyte has ∼6 × 10^4^ copies of the genome, indicating that some *Carsonella* cells can contain thousands or even tens of thousands of genomic copies per cell. The basis of polyploidy of *Carsonella* is unknown, but it potentially plays a role in the repair of DNA damage through homologous recombination.

**IMPORTANCE** Mitochondria and plastids are endosymbiotic organelles in eukaryotic cells and are derived from free-living bacteria. They have many highly reduced genomes from which numerous genes have been transferred to the host nucleus. Similar, but more recently established, symbiotic systems are observed in some insect lineages. Although the genomic sequence data of such bacterial symbionts are rapidly accumulating, little is known about their ploidy. The present study revealed that a bacterium with a drastically reduced genome is an extreme polyploid, which is reminiscent of the case of organelles.

## OBSERVATION

Polyploidy, the state where organisms have multiple copies of the genome within a nucleus or a cell, has repeatedly evolved across the domains of life (1). Whereas most bacteria are monoploid or mero-oligoploid only during fast growth, some species, including cyanobacteria and extremophiles, are stably polyploid (1–4). Mitochondria and plastids, endosymbiotic organelles that are derived from free-living bacteria, have highly reduced genomes and are also polyploid (5, 6). In this context, the ploidy of vertically transmitted organelle-like symbionts of insects attracts our interest. Various insect lineages harbor phylogenetically diversified bacterial symbionts within the specialized cells called bacteriocytes, which constitute the bacteriome organ (7). The bacteriome-associated symbionts have drastically reduced genomes like organelles (7), and their polyploidy has generally been suspected based on the strong signal intensity of fluorescent DNA staining. However, quantitative analyses of their ploidy have been reported for only two species thus far (8–10). Dot-blot hybridization, fluorimetry, and quantitative PCR showed that Buchnera aphidicola (Gammaproteobacteria, Enterobacterales; genome size: 640 kb) (11) of the pea aphid, Acyrthosiphon pisum (Hemiptera, Aphidoidea), had 10 to 600 genomic copies per cell depending on developmental stages and morphs of the host insect (8, 9). Digital PCR using four individual cells showed that Candidatus Sulcia muelleri (Bacteroidetes; genome size, 240 kb) of the green sharpshooter Draeculacephala minerva (Hemiptera, Membracoidea) had 200 to 900 genomic copies per cell (10).

**FIG 1:**
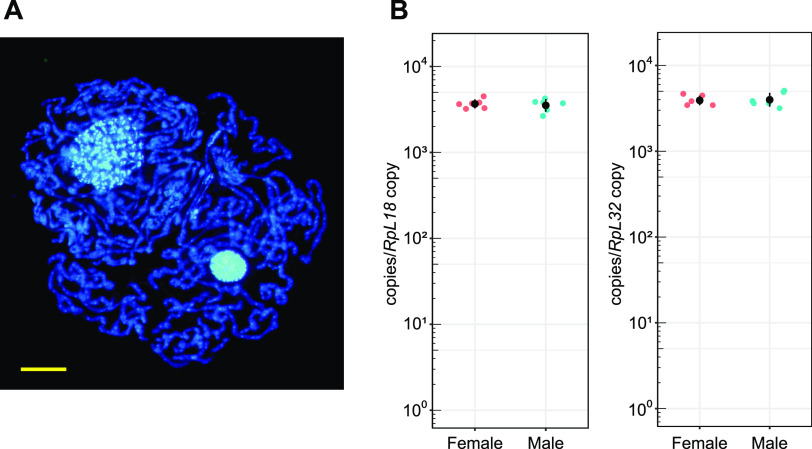
(A) Bacteriocytes stained with DAPI. Tubular *Carsonella* cells were squeezed out by applying gentle pressure on a coverslip. Extremely long *Carsonella* cells surrounding host nuclei show strong DAPI signals. Within *Carsonella* cells, numerous spots with higher signal intensities are observed. Bar, 10 μm. (B) Quantitative PCR analysis of *Carsonella* genomic copy number in the bacteriome of Pachypsylla venusta. Abundance values of the *Carsonella* 16S rRNA gene were normalized to the psyllid nuclear genes for RpL18 (left) and RpL32 (right). All data points (female, magenta; male, cyan) of six biological replicates of each sex are presented. Black dots and black bars represent means and standard deviations, respectively.

Candidatus Carsonella ruddii (Gammaproteobacteria, Oceanospirillales) (Fig. 1A), the obligate symbiont of the hackberry petiole gall psyllid, Pachypsylla venusta (Hemiptera, Psylloidea), has a genome of 160 kb, one of the smallest genomes known for cellular organisms (12). To measure the ploidy of bacteriome-associated symbionts, we performed absolute quantitative real-time PCR to estimate the ploidy of *Carsonella* harbored in uninucleate bacteriocytes (13–15). Whereas most psyllid species have another (secondary) bacterial symbiont in a syncytial region within the bacteriome (13, 16–18), P. venusta lacks secondary symbionts and the syncytium is rudimentary (12, 15, 19). Thus, we extracted DNA from the whole bacteriome isolated from male and female nymphs of P. venusta for use as templates in quantitative PCR. To assess copy numbers of the *Carsonella* genome, the 16S rRNA gene, a single-copy gene encoded in the *Carsonella* genome (12), was amplified with specific primers (Table 1). For calibration, the genes encoding ribosomal protein (Rp)L18 and RpL32, which are single-copy genes in the P. venusta genome (20), were also quantified. The results showed that the copy number of the 16S rRNA gene per copy of the *RpL18* gene was 3690 ± 460 (mean ± standard deviation, *n* = 6) for females and 3571 ± 575 (*n* = 6) for male insects. When calibrated with the *RpL32* gene, the values were 3942 ± 518 (*n* = 6) for females and 4040 ± 769 (*n* = 6) for male specimens (Fig. 1B). Because the bacteriocytes of P. venusta are 16-ploid (15), these data indicate that *Carsonella* within a single bacteriocyte has ∼6 × 10^4^ copies (16 × ca. 4 × 10^3^ copies) of the genome, assuming that all bacteriocytes are uninucleate. Because *Carsonella* is pleomorphic and usually tubular, the cell number within a single bacteriocyte varies (12, 14, 18). In some cases, *Carsonella* can be extremely long (greater than hundreds of micrometers), and only a few *Carsonella* cells are observed within a single bacteriocyte (Fig. 1A). Thus, a *Carsonella* cell can contain thousands or even tens of thousands of genomic copies. This is much more than observed in *Buchnera* or *Sulcia* (8–10) and analogous to the case of *Epulopiscium* sp. (Firmicutes), a giant bacterium observed in the fish gut, which contains tens of thousands of genomic copies per cell (21). There appears to be a tendency that bacteria with large cell sizes to be highly polyploid, as evidenced by a recent report that Candidatus Thiomargarita magnifica, a centimeter-long bacterium, has half a million copies of the genome (22).

**TABLE 1:** Gene-specific primers used in this study

Target gene	Primer	Sequence	Product size
*Carsonella* 16S rRNA	CRPV_16S_10F	CATAGCTCAGATTGAACGCTGGTA	89
	CRPV_16S_98R	CTCACCCGTTCGCTGCTAATAC	
P. venusta *RpL18*	PV_rpL18_420F	AGAACTGGACGAGAAGCCAACA	81
	PV_rpL18_500R	TGATCTGACGTGTGCTTTTGTATG	
P. venusta *RpL32*	PV_rpL32_359F	GGTCACGCTGTGTCTTCAAA	85
	PV_rpL32_443R	GGGCATGTCCATTGGTAAGT	

A potential cause of the polyploidy of *Carsonella* is mutational degradation of ancestral genes linking chromosome replication to cell division, resulting in extremely large cell sizes and polyploidy. However, a presumed benefit of polyploidy is to facilitate the mutualistic role of synthesizing essential amino acids that are required by the host psyllid (12) because polyploidy is assumed to increase metabolic output, diminishing the need to allocate resources and energy to cell division (1, 23). Additionally, the ploidy of *Carsonella* may have an evolutionary impact. Like other bacteriome-associated obligate symbionts of insects, *Carsonella* is confined in the bacteriocyte, and only a small part of the maternal population is transovarially transmitted to the next generation (18). This type of small, bottlenecked, and asexual population suffers from the accumulation of mildly deleterious mutations through the process known as Muller’s ratchet (24). Polyploidy could mask fresh mutations, and facilitate conventional repair processes within cells, such as the repair of double-strand breaks. However, for polyploidy to slow Muller’s ratchet, nonmutant sequences must be disproportionately favored as repair templates. A role of polyploidy in facilitating within-cell DNA repair is consistent with the conservation of *recA*, encoding the central repair enzyme, in sequenced *Carsonella* genomes (12, 16, 17, 25), though this role may not extend to all bacteriome-associated symbionts, many of which lack *recA* (7). Thus, further studies are required to assess ploidy and its evolutionary role in these symbionts.

## MATERIALS AND METHODS

### Absolute quantitative real-time PCR.

Absolute quantification was performed by real-time quantitative PCR targeting the *Carsonella* 16S rRNA gene and the P. venusta genes for RpL18 and RpL32. Galls containing 5th instar nymphs of the hackberry petiole gall psyllid, Pachypsylla venusta, were collected from hackberry trees, Celtis reticulata, in Tucson, AZ. Insects were removed from galls and dissected under a stereomicroscope. Individuals were sexed based on gonads therein, and bacteriomes were isolated and pooled from 3 to 5 individuals of either sex. DNA was extracted from the pooled bacteriomes using DNeasy blood and tissue kit (Qiagen). The quality of extracted DNA was assessed using a NanoDrop 2000c spectrophotometer (Thermo Fisher Scientific) and the quantity was assessed using a Qubit 2.0 Fluorometer with a Qubit dsDNA HS assay kit (Thermo Fisher Scientific). Reference standards for quantification were constructed by PCR followed by TA-cloning as described previously (26). Briefly, PCR was performed using genomic DNA extracted from P. venusta and gene-specific primers (Table 1). The PCR products were cloned into the pGEM-T Easy vector (Promega) and amplified in Escherichia coli JM109. Inserts were sequenced following colony PCR using M13-F (5′-GTAAAACGACGGCCAG-3′) and M13-R (5′-CAGGAAACAGCTATGAC-3′) primers annealing to the vector. Plasmids with appropriate inserts were amplified in E. coli and purified using a Fast Plasmid Minikit (Eppendorf). PCRs were performed again using purified plasmids and M13-F and M13-R primers. Subsequently, PCR products were purified with a QIAquick PCR purification kit (Qiagen) and quantified with a Qubit 2.0 Fluorometer and Qubit dsDNA BR assay kit (Thermo Fisher Scientific). Copy numbers of the PCR products were calculated based on their concentration and molecular weights. For each target gene, 10^8^, 10^7^, 10^6^, 10^5^, 10^4^, and 10^3^ copies/μL of PCR product solutions were freshly prepared in LoBind tubes (Eppendorf) for use as reference standards. Real-time quantitative PCR was performed using the LightCycler instrument and FastStart DNA Master SYBR green I kit (Roche). Running parameters were 95°C for 10 min, followed by 40 cycles of 95°C for 10 s, 58°C for 3 s, and 72°C for 6 s. Signal intensity was measured at the end of each elongation phase. The absence of nonspecific products was confirmed by melting curve and electrophoretic analyses. Copy numbers of target genes in specimens were calculated based on standard curves generated with reference standards using the LightCycler software (ver. 3.0, Roche). The copy number of the *Carsonella* 16S rRNA gene was normalized to the copy numbers of the P. venusta genes for RpL18 and RpL32. Analyses were performed with six biological replicates for each sex and three technical replicates.

### Microscopy.

Bacteriomes were dissected from 5th instar nymphs, fixed with fixation buffer (1% glutaraldehyde, 20 mM Tris-HCl [pH 7.65], 2.5 mM EDTA, 3.2 mM spermidine, 7 mM 2-mercaptoethanol, 0.4 mM phenylmethyl-sulfonyl fluoride), and stained with 4′,6-diamidino-2-phenylindole (DAPI). After repetitive pipetting, specimens were put on a glass slide and covered with a coverslip. The slides were examined by fluorescence microscopy (BX-53; Olympus).
